# Vesicular glutamate transporter 2 expression in the ventral tegmental area of outbred male rats following exposure to nicotine and alcohol

**DOI:** 10.1016/j.dadr.2023.100180

**Published:** 2023-07-13

**Authors:** Maria Vrettou, Stefan Bernhard Thalhammer, Anne-Lie Svensson, Sylvie Dumas, Kent W Nilsson, Åsa Wallén-Mackenzie, Robert Fredriksson, Ingrid Nylander, Erika Comasco

**Affiliations:** a Department of Women's and Children's Health, Science for Life Laboratory, Uppsala University, Uppsala, Sweden; b Department of Pharmaceutical Biosciences, Uppsala University, Uppsala, Sweden; c Oramacell, Paris, France; d Centre for Clinical Research Västerås, Uppsala University, Västmanland County Hospital Västerås, Sweden; e Department of Organismal Biology, Uppsala University, Uppsala, Sweden

**Keywords:** alcohol, brain, expression, nicotine, *Vglut2*

## Abstract

**Background:**

Initiation of use/co-use of nicotine and alcohol, commonly occurring in an episodic manner during adolescence, can imprint vulnerability to the developing brain and lead to addiction. The ventral tegmental area (VTA) is a key heterogeneous region of the mesocorticolimbic circuit involved in the binge-drinking and intoxication step of the addiction circuit. Higher human post-mortem VTA expression of *vesicular glutamate transporter 2 (VGLUT2),* a marker of the glutamatergic phenotype also expressed in dopaminergic [Tyrosine Hydroxylase (Th)-positive] neurons, has been associated with chronic nicotine use and co-use with alcohol.

**Methods:**

The present study aimed to map and characterize the *Vglut2*- and *Th-*expressing neurons in the VTA of adolescent male rats exposed or not to prolonged (six-weeks) episodic (three consecutive days/week) nicotine and/or alcohol administration. Nicotine (0.35 mg/kg free base) was injected subcutaneously, whereas alcohol (2 g/kg 20%) was administrated via gavage. *Vglut2* and *Th* mRNA was assessed in the anterior and posterior VTA by use of *in situ* hybridization.

**Results:**

The profile of neurons varied with substance-exposure among VTA subregions. *Th-*only expressing neurons were more abundant in the posterior VTA of the group exposed to nicotine-only, compared to controls. The same neurons were, on the contrary, less present in the anterior VTA of animals exposed to alcohol-only, who also displayed a higher number of *Vglut2-*expressing neurons in the lateral anterior VTA.

**Conclusions:**

VTA *Vglut2-* and *Th*-only neurons seem differentially involved in the effects of adolescent episodic nicotine and alcohol exposure in the anterior and posterior VTA.

## Highlights

• More dopaminergic-only neurons upon nicotine in the posterior VTA.
• Less dopaminergic-only neurons upon alcohol in the anterior VTA.
• More glutamatergic neurons in the lateral anterior VTA.

## 1 Introduction

Initiation of alcohol and nicotine use typically occurs during adolescence, with 33% and 18% of adolescents in Europe at 15-16 years of age reporting to have used alcohol or smoked tobacco cigarettes, respectively, before the age of thirteen, i.e. early onset (ESPAD, 2020). Moreover, co-use of both substances is high, with 93% of the adolescents who have used tobacco cigarettes at least once, have also used alcohol. On the other hand, 54% of those who have consumed alcohol have also used tobacco cigarettes (Kraus, 2015). Additionally, binge-drinking and weekend-like nicotine use are highly associated with the adolescent phase (Harrison et al., 2008). Early onset, and such patterns, of substance use and misuse can influence brain development, consequently increasing the risk for psychopathology, including Alcohol Use Disorder (AUD) (Dawson et al., 2008) and Tobacco Use Disorder (TUD) (Camenga & Klein, 2016). Yet, the shared neurobiological underpinnings of co-use and comorbidity are not fully understood, especially during adolescence.

Nicotine exerts its effect via activation of widely distributed nicotinic acetylcholine receptors (nAChRs), which in turn stimulate the release of various neurotransmitters, among others, dopamine and glutamate (Yan et al., 2018). Alcohol interacts with several receptors and ion channels, including nAChRs (Marszalec et al., 1999), present on dopaminergic and glutamatergic neurons of the mesocorticolimbic pathways (Adams, 2017; Jones & Wonnacott, 2004). Both alcohol and nicotine lead to a dopaminergic overload (Di Chiara & Imperato, 1988) in the nucleus accumbens (Acb) through projections from the ventral tegmental area (VTA), a key region of the mesocorticolimbic system involved in reward and reinforcing effects of these two drugs (Koob & Volkow, 2010). The VTA has recently been profiled as more heterogeneous than previously believed based on several parameters, including afferent and efferent projections, electrophysiological profiles, molecular properties, role in behavioral regulation and not least neurotransmitter identity. While long assumed to be composed of dopamine neurons only, it is now known that glutamatergic and GABAergic neurons co-exist with dopamine neurons, and that many VTA neurons co-transmit more than one of these neurotransmitters (Eskenazi et al., 2021; Morales & Margolis, 2017). Furthermore, distinct functions of the anterior (aVTA) and the posterior VTA (pVTA) have been demonstrated (Sanchez-Catalan et al., 2014). Although addictive drugs have been shown to be readily self-administered in the pVTA (Ikemoto, 2007), only the aVTA has been linked to nAChR-mediated alcohol-induced elevation of dopamine in the Acb (Ericson et al., 2008).

Owing to its modulatory role on the mesocorticolimbic dopaminergic system, the glutamatergic pathway stemming from the midbrain and projecting to the Acb has been consistently implicated in various phases of the addiction cycle (D'Souza, 2015), being involved in both rewarding and aversive behaviors (Qi et al., 2016; Wang et al., 2015). The glutamatergic identity can be defined by the presence of vesicular glutamate transporters (VGLUTs), which act as transporters of cytosolic glutamate into presynaptic vesicles and enable the exocytotic release of glutamate. Hence, the presence of VGLUTs defines a neuron's ability to use glutamate as neurotransmitter (Fremeau et al., 2001, Wallén-Mackenzie et al., 2009). Expression of the *Vglut2* gene (*Slc17a6*) in the maturing and adult brain is found in neurons of the VTA, particularly the medial VTA (Fremeau et al., 2001), both in classical glutamatergic neurons, and in dopaminergic [*Tyrosine Hydroxylase (Th)-*positive] neurons that co-release dopamine together with glutamate (El Mestikawy et al., 2011, Trudeau et al., 2014). These VTA neuronal subtypes project to, and modulate, key reward regions, the Acb and the prefrontal cortex, in different ways and intensities (Aguilar et al., 2017; Papathanou et al., 2018; Yamaguchi et al., 2011).

VGLUT2*-*mediated neurotransmission from midbrain dopamine neurons has been consistently highlighted in addiction-related phenotypes (Bimpisidis and Wallen-Mackenzie, 2019, Eskenazi et al., 2021). For example, mice lacking *Vglut2* gene expression in midbrain dopamine neurons show reduced locomotor response upon acute amphetamine and cocaine administration (Birgner et al., 2010; Hnasko et al., 2010). Further, higher cocaine consumption and cocaine-cue-induced seeking behavior were observed in a self-administration paradigm (Alsiö et al., 2011). Moreover, ablation of *Vglut2* gene expression in mature dopamine neurons has been shown to contribute to decreased glutamatergic neurotransmission and synaptic strength in the Acb, while it did not affect locomotory response to psychostimulants (Papathanou et al., 2018). In addition, an elevated expression profile of the *Vglut2* gene has been demonstrated in the VTA of alcohol-consuming rats previously exposed to early-life stress (Vrettou et al., 2017). In humans, *VGLUT2* genotype was shown to be associated with alcohol dependence (Comasco et al., 2014), as well as with moderate susceptibility to the environment in relation with alcohol-related problems among adolescents and young adults (Vrettou et al., 2019).

Altogether this suggests a role of VGLUT2 in reward-related functions of relevance to various aspects of addiction, pointing to a model in which glutamate/dopamine co-release by midbrain neurons plays an intricate role in reward responsiveness and behavioral reinforcement mediated by dopamine (Bimpisidis and Wallen-Mackenzie, 2019, Eskenazi et al., 2021). To date, one study has investigated the association between the use of nicotine (alone and in combination with alcohol) and *VGLUT2* expression in the VTA of human post-mortem brains (Flatscher-Bader et al., 2008). Compared to controls, *VGLUT2* expression in the VTA was higher among persons with both AUD and TUD, and even higher among individuals with TUD only (Flatscher-Bader et al., 2008). Furthermore, neonatal nicotine exposure in mice increased the number of VTA *Vglut2^pos^/Th^pos^* neurons and nicotine preference in adulthood (Romoli et al., 2019). Whether VTA glutamate-related signatures of both nicotine and alcohol consumption emerge already in the initial stages of use in previously drug-naïve individuals remains to be investigated.

The present study aimed to quantify and characterize *Vglut2-*expressing neurons in the VTA of adolescent outbred male rats following exposure to episodic intake of nicotine, alone or together with alcohol. The hypothesis was that rats exposed to nicotine alone, or in combination with alcohol, would show a higher number of *Vglut2-*expressing neurons in the VTA, as previously shown in persons with substance use disorder (Flatscher-Bader et al., 2008). Furthermore, the distinct cellular profile in the VTA, containing a larger population of *Vglut2^pos^/Th^neg^* and *Vglut2^neg^/Th^pos^*, and a smaller population of *Vglut2^pos^/Th^pos^*, was hypothesized to differentially contribute to any relevant variations, and was thus further investigated.

## 2 Materials and methods

### 2.1 Animals

Twenty time-mated females (Harlan Laboratories B.V., Horst, the Netherlands) were received into the lab on gestation day 15. The dams were housed individually in standard cages (59 × 38 × 20 cm) under normal light-dark cycle having water and food *ad libitum*. On the day of birth (postnatal day (PND) 0), the litters were cross-fostered and mixed so as to avoid biological littermates. On PND 21 the pups were weaned and group-housed (2-3 per cage) under standard conditions (22°C, 50 ± 10% humidity) in reversed light-dark cycle having access to water and pellet food *ad libitum*. Due to limited resources, only male rats were included in the study. All animal experiments were approved by the Uppsala Animal Ethical Committee (C427/12) and followed the Guide for the Care and Use of Laboratory Animals and the guidelines of the Swedish Legislation on Animal Experimentation (Animal Welfare Act SFS1998:56) and the European Communities Council Directive (86/609/EEC).

### 2.2 Adolescent nicotine and/or alcohol exposure

**Fig. 1:**
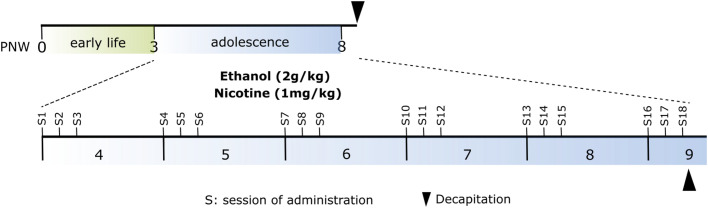
Outline of the experimental model. Four experimental groups (control, ethanol, nicotine and ethanol and nicotine; n = 10 animals per group) were included. Administration of ethanol (2g/kg 20%) and nicotine (0.35 mg/kg free base) took place for three consecutive days per week for six weeks during adolescence.

Forty outbred male Wistar rats were randomly assigned to four experimental groups (n = 10, per group) exposed to either: i) nicotine-only (water and nicotine); ii) alcohol-only (alcohol and saline); iii) combination of nicotine and alcohol; or iv) control (water and saline) for three consecutive days per week for six weeks during adolescence [postnatal week (PNW) 4-9]. Administration was given at 09:00 am. Alcohol (Solveco Etanol A 96%, Solveco AB, Rosersberg, Sverige) was diluted in water and nicotine [(−)-Nicotine hydrogen tartrate salt, Sigma Aldrich] in saline. Alcohol and water were administrated via gavage whereas nicotine and the control saline solution were injected subcutaneously. This exposure paradigm was chosen to mimic episodic drug intake commonly used among adolescents, based on the literature as reviewed by (Carnicella et al., 2014; Sanchis-Segura & Spanagel, 2006). The alcohol dose (2 g/kg 20%) was chosen to achieve blood alcohol levels > 0.08g/dl (Lundberg, 2020) (further supported by unpublished data) and the nicotine dose (0.35 mg/kg free base) was based on previous literature (Lof et al., 2007). Higher doses were not chosen since the aim was to mimic adolescent use and to render an adequate intoxication level in the rat (Lundberg, 2020). At PNW 9, the animals were decapitated two hours after the last session of drug administration and the whole brain was collected and stored in -80°C until further analysis (Fig. 1).

### 2.3 Tissue collection

The brain of each animal was removed and immediately immersed in iso-pentane, kept around -20°C on dry ice, for 2 minutes, and was then stored at -80°C until further analysis. Coronal cryosections of 14µm were collected on Superfrost slides (Menzel-Gläser, Braunschweig, Germany) from bregma -4.80 to -6.84mm, targeting the VTA, including the aVTA (defined as -4.8 to -5.2mm from bregma, and the pVTA (defined as caudal to -5.3mm from bregma), according to Paxinos and Watson, 6th edition (Paxinos G., 2007), using the Cryostar NX70 cryostat (Thermofisher Scientific, Waltham, MA, USA), and stored at -80°C until further analysis.

### 2.4 Double Fluorescent *in situ* Hybridization (d-FISH) and FISH/Chromogenic ISH (CISH)

Double FISH (aVTA) or combined FISH/CISH (pVTA) was performed using RNA probes to determine neurons expressing *Slc17a6/Vglut2* (NM_080853.3, sequence 2315-3244) and/or the dopaminergic marker tyrosine hydroxylase (TH), encoding the rate-limiting enzyme in the production of dopamine, (*Th,* NM_012740.3, sequence 456-1453) in the rat brain. Riboprobes were synthesized with DIG- or fluorescein-labeled ribonucleoside tri-phosphate. dFISH and FISH/CISH were carried out as previously described (Bimpisidis et al., 2019) on cryosections that were air-dried, fixed in 4% paraformaldehyde and acetylated in 0.25% acetic anhydride/100 mM triethanolamine (pH 8). Sections were hybridized for 18h at 65°C in 100µl of formamide-buffer containing 1µg/ml *Slc17a6* DIG-labeled and 1µg/ml *Th* fluorescein-labeled riboprobe. They were then washed at 65°C with Saline Sodium Citrate (SSC) buffers of decreasing strength and blocked with 20% fetal bovine serum and 1% blocking solution. Fluorescein epitopes were detected with horseradish peroxidase (HRP) conjugated anti-fluorescein antibody at 1:5000 and revealed using Cy2-tyramide at 1:250. HRP-activity was stopped by incubation of sections in 0.1M glycine followed by a 3% H2O2 treatment. DIG epitopes were detected with HRP anti-DIG Fab fragments at 1:3000 (Roche, Mannheim, Germany) and revealed using Cy3 tyramide at 1:100 (dFISH) or alkaline phosphatase-coupled anti-DIG Fab fragments at 1/1000 (Roche, Mannheim, Germany) and developed with NBT/BCIP 1/100 (Roche, Mannheim, Germany) (FISH/CISH). Nuclear staining was performed with 4′ 6-diamidino-2-phenylindole (DAPI).

### 2.5 Cell imaging and counting

FISH and FISH/CISH stained slides were imaged using the Hamamatsu Nanozoomer 2.0-HT (Hamamatsu Photonics, Hamamatsu City, JPN). The whole slides were scanned as one batch with the same settings and a 20x objective, and the final images were saved in NDPI format. Using NDP.view 2 brightness, contrast and saturation of the individual channels were identically adjusted for all images depending on the detection technique, i.e. dFISH or FISH/CISH.

Image-masking with the respective region of interest (ROI) of the corresponding bregma was performed by alignment of the figures of the rat brain atlas, Paxinos and Watson, 6th edition (Paxinos G., 2007) on Inkscape (version 0.92) as a template with specific anatomical reference points. The ROIs in the VTA were bregma -4.92mm (aVTA); and bregma -5.3/-5.4mm (pVTA). Additionally, the sub-nuclei of the aVTA [parabrachial pigmented nucleus (PBP), VTA rostral nucleus (VTAR) and rostral linear nucleus (RLi), and of the pVTA (PBP, RLi, paranigral nucleus (PN) and interfasicular nucleus (IF)] (Paxinos G., 2007) were analyzed separately.

A standardized staining signal identification was performed using CellProfiler (version 3.1.5) (Carpenter et al., 2006) through recognition of objects on the basis of size, shape, intensity, and texture of the signal in the dFISH images (aVTA) (Supplemmentary Material). Cell counting was performed manually for FISH/CISH images (pVTA), in ImageJ (Schindelin et al., 2015), as due to the presence of different channels (i.e. brightfield and fluorescent) in the same image and the general low quality of staining, the already developed pipeline for aVTA, was not applicable herein. Manual counting was performed three times, independently and in a blind manner. In each ROI, the count of the neurons expressing *Vglut2* and/or *Th* to the associated nuclei was determined*,* i.e. *Vglut2-*positive/*Th*-negative (*Vglut2^pos^/Th^neg^*)*, Vglut2-*negative/*Th*-positive (*Vglut2^neg^/Th^pos^*) and *Vglut2-*positive/*Th*-positive (*Vglut2^pos^/Th^pos^*), and was used to estimate the percentage of each neuronal population vs. the total number of DAPI-stained nuclei within a ROI. Lastly, the percentage of all *Vglut2^pos^* neurons vs. the total number of DAPI-stained nuclei was generated by summation of both the *Vglut2^pos^/Th^neg^* and *Vglut2^pos^/Th^pos^* populations.

### 2.6 Data analysis

Normality of data distribution was assessed with Shapiro-Wilk test. Some samples were excluded from further analyses due to technical problems, including bad staining quality, nicked slices, and incorrect bregma. The final sample size for each group analyzed for the aVTA was: control: n = 10; nicotine: n = 9; alcohol: n = 8; nicotine and alcohol: n = 10; and for the pVTA: control: n = 5; nicotine: n = 7; alcohol: n = 5; nicotine and alcohol: n = 8. For each ROI, two-way ANOVA was used to test the interactive effect between nicotine and alcohol on the percentage of *Vglut2^pos^* cells, as well as the three different neuronal subpopulations. The two-way ANOVA was followed by simple main effects analysis to asses between group differences on the percentage of *Vglut2* and *Th* neuronal subpopulations. Analyses were performed using the SPSS software (IBM SPSS Statistics for Windows, Version 25.0. Armonk, NY: IBM Corp). The datasets generated and/or analysed during the current study are available from the corresponding author upon reasonable request.

## 3 Results

### 3.1 *Vglut2* expression and neuronal subpopulations in the control group

The *Vglut2* expression pattern in the control group was in accordance with previously demonstrated mRNA expression profiles in the rat VTA (Papathanou et al., 2018; Yamaguchi et al., 2007, Yamaguchi et al., 2011). In both the anterior and the posterior VTA, the ratio of *Vglut2-* expressing neurons was the highest in the RLi (anterior: mean = 46 % ± 9; posterior: mean = 12.8 % ± 6.2) and the lowest in the PBP (anterior: mean = 13 % ± 4.7; posterior: mean = 6.2 % ± 2). The relative percentage (to DAPI-stained nuclei) of *Vglut2-*expressing neurons was 41.3% ± 6 (mean ± SD) in the VTAR (aVTA), 10.9% ± 4.8 (mean ± SD) in the PN (pVTA) and 9.9 % ± 5.1 (mean ± SD) in the IF (pVTA), confirming previous reports of a lateromedial increasing gradient of *Vglut2* distribution, as well as a decreasing gradient of distribution along the rostro-caudal axis (higher in the aVTA and lower towards the pVTA) (Yamaguchi et al., 2011).

**Fig. 2:**
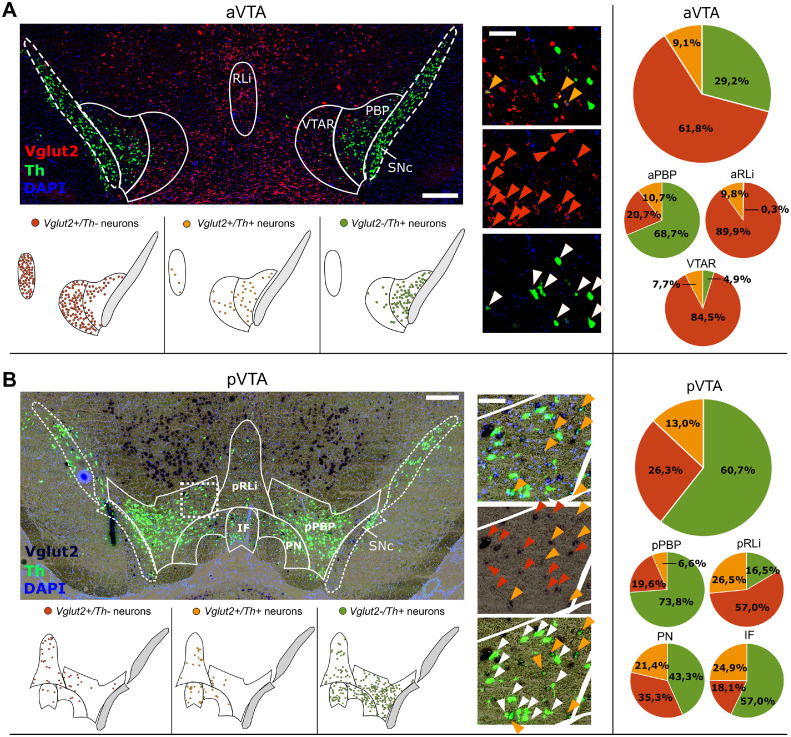
In situ and schematic distribution (left panel) and percentages (right panel) of the neuronal subpopulations Vglut2^pos^/Th^neg^ (red), Vglut2^neg^/Th^pos^ (green), and Vglut2^pos^/Th^pos^ (orange) in the (A) aVTA and subregions (bregma -4.92) and (B) pVTA and subregions (bregma -5.4), in the control group; scale bar: 400µm, inset: 100µm. Yellow arrows in the insets represent co-expression of Vglut2 and Th, red arrows represent Vglut2 expression, and white arrows Th expression. All of the VTA subregions express the Vglut2 gene, though in a decreasing gradient of concentration along the rostro-caudal axis (Yamaguchi et al., 2007). Pronounced Vglut2 expression is observed from bregma -4.92 to -5.5mm, with a higher expression in the midline compared to lateral subregions (Yamaguchi et al., 2011).

The relative percentage (to DAPI-stained nuclei) of the three investigated neuronal subtypes (*Vglut2^pos^/Th^neg^, Vglut2^neg^/Th^pos^, Vglut2^pos^/Th^pos^*) in the VTA and its subregions in the control group is shown in Fig. 2. In the aVTA, the relative percentage of neurons co-expressing *Vglut2* and *Th* (*Vglut2^pos^/Th^pos^*) was similar throughout the subregions (7.7% — 10.7%), while *Vglut2^neg^/Th^pos^* neurons were predominant in the aPBP, and *Vglut2^pos^/Th^neg^* neurons were the majority in the aRLi and VTAR. On the contrary, in the pVTA, the percentage of *Vglut2^pos^/Th^pos^* neurons varied among the subregions; they were highest in the pRLi and IF, and lowest in the pPBP, in which *Vglut2^neg^/Th^pos^* neurons were predominant instead, while *Vglut2^pos^/Th^neg^* neurons were the majority in the pRLi.

### 3.2 Effect of nicotine and/or alcohol exposure on *Vglut2-*expression and *Vglut2 /Th* neuronal subpopulations

**Fig. 3:**
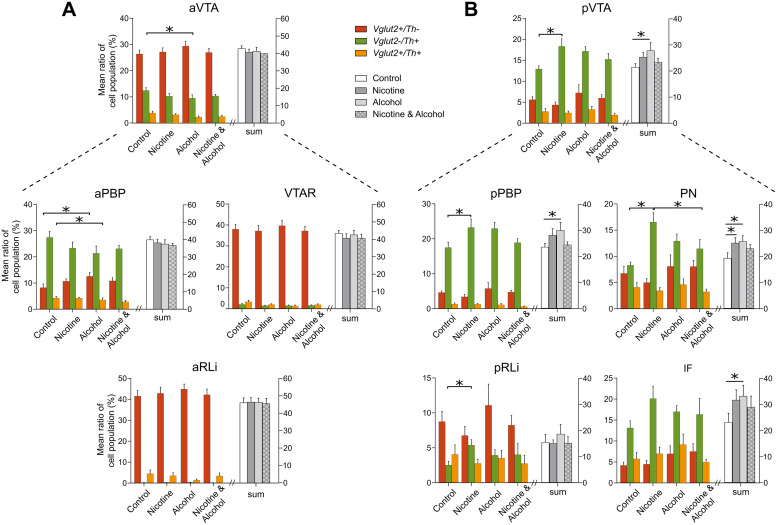
Percentages of Vglut2^pos^/Th^neg^, Vglut2^neg^/Th^pos^, and Vglut2^pos^/Th^pos^ neurons, and their sum, in the a) aVTA and subregions and b) pVTA and subregions among groups. Percentages of each neuronal population were estimated by computing the number of neurons expressing Vglut2 and/or Th vs. the total number of the associated DAPI-stained nuclei within a region of interest *: p ≤ 0.05; error bars = 1SE IF: interfasicular nucleus; PBP: parabrachial pigmented nucleus; PN: paranigral nucleus; RLi: rostral linear nucleus; VTA: ventral tegmental area; VTAR: VTA rostral nucleus

In the aPBP, there was an interactive effect on the relative percentage of *Vglut2*-only positive neurons (F _(1,32)_ = 4.479; *p* = 0.042), driven by the higher % of *Vglut2*-only neurons upon alcohol exposure (*p* = 0.017) compared to the controls (Fig. 3a). No group differences were seen in the relative number of *Vglut2^pos^* or *Vglut2^pos^/Th^neg^* neurons (%) in the pVTA (Fig. 3b).

Based on the suggested involvement of *Vglut2*-expressing dopaminergic neurons (*Vglut2^pos^/Th^pos^*) of the VTA in addictive behaviour (Alsiö et al., 2011, Bimpisidis and Wallen-Mackenzie, 2019; Birgner et al., 2010; Hnasko et al., 2010), the effect of the drugs on the neurons co-expressing *Vglut2* and *Th* was further investigated*.* A main effect of alcohol was seen in the aPBP towards lower number (%) of the *Vglut2^pos^/Th^pos^* neurons (F _(__1,32)_ = 7.408; *p* = 0.01). There was no difference in the number of *Vglut2^pos^/Th^pos^* (%) as a result of nicotine and/or alcohol exposure in any other subregion of the aVTA or in the pVTA (Fig. 3).

**Fig. 4:**
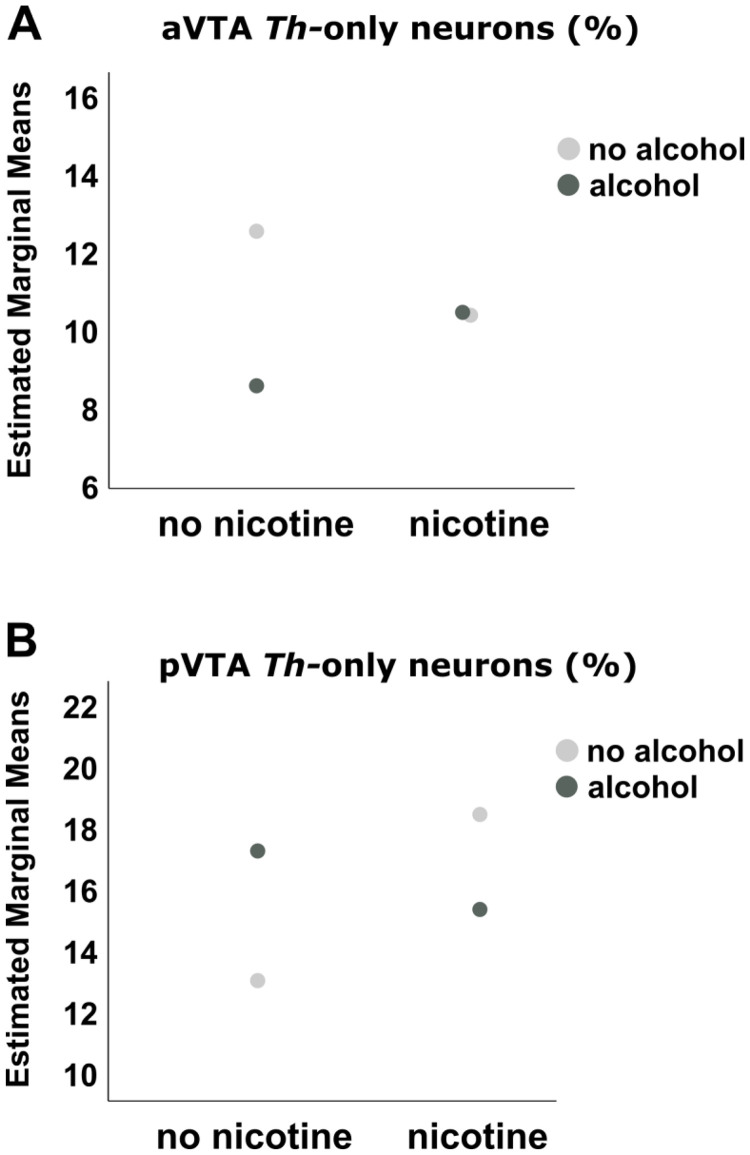
Depiction of the interaction between nicotine and alcohol on the percentage of Vglut2^neg^/Th^pos^ neurons to DAPI-stained cells in the a) aVTA and b) pVTA.

In the pVTA, there was an interactive effect between nicotine and alcohol on the ratio (%) of *Vglut2^neg^/Th^pos^* neurons (F _(1,21)_ = 6.118; *p* = 0.022). The ratio (%) of *Vglut2^neg^/Th^pos^* neurons was higher in the group exposed to nicotine-only (*p* = 0.019), whereas no effect of combination of both drugs was observed (Figs. 3b and 4b).

The same effect was seen in the pPBP (F *_(1,21)_* = 6.924; *p* = 0.016), driven by the higher number (%) of *Th*-only positive neurons upon nicotine (*p* = 0.043) compared to controls. An interactive effect was observed also in the PN (F _(1,21)_ = 8.469; *p* = 0.008), driven by a higher number (%) of *Th*-only positive neurons upon nicotine (*p* = 0.002) compared to controls, and lower (%) *Th*-only positive neurons upon the combination of drugs (*p* = 0.025) compared to nicotine-only group.

In the aVTA, an interactive effect between nicotine and alcohol towards the opposite direction was found on the *Vglut2^neg^/Th^pos^* neurons (F _(1,32)_ = 4.665; *p* = 0.038), driven by a lower number (%) of *Th*-only positive neurons upon alcohol exposure (*p* = 0.007) compared to the controls (Fig. 4a). Non-significant findings on the trend level, as well as interactive effects between region (anterior vs posterior VTA) and group on the three different neuronal populations, are reported in the Supplementary Material.

### 3.3 Effect of nicotine and/or alcohol exposure on the sum (%) of *Vglut2-* and *Th-* neuronal subpopulations

Similar effects of the drugs were seen on the percentage of the sum of all cell populations (*Vglut2^pos^/Th^neg^, Vglut2^neg^/Th^pos^, Vglut2^pos^/Th^pos^*)/DAPI (Fig. 3). In the pVTA, an interactive effect between nicotine and alcohol was seen on the percentage of the sum (F _(1,21)_ = 4.287; *p* = 0.05), which was higher in the alcohol-only group (27.7 ± 6.9; *p* = 0.05) compared to the control group (21.3 ± 3). This was driven by the PBP (F _(1,21)_ = 5.004; *p* = 0.036), and the PN (F _(1,21)_ = 4.479; *p* = 0.046), and by the *Vglut2^neg^/Th^pos^* population. In the aVTA, no difference was found in the sum of cells between the control and the treated groups (Fig. 3a).

## 4 Discussion

*Vglut2-* and *Th-*expressing neurons were assessed in the VTA of non-preferring rats (i.e. not selectively bred for nicotine or alcohol preference) in relation to prolonged nicotine and/or alcohol administration during adolescence. Higher percentage of *Vglut2^pos^/Th^neg^* neurons was found in the lateral aVTA, and especially the PBP, of the animals exposed to alcohol-only compared to controls. The present findings do not corroborate the ones on human post-mortem brains, in which higher *VGLUT2* expression was observed in the VTA of individuals with TUD-only and both TUD and AUD, but not individuals with AUD-only, compared to controls (Flatscher-Bader et al., 2008). Nevertheless, it is important to note that the model used here (i.e. drug-naïve, non-addicted, non-preferring adolescent rats), cannot be directly compared with the aforementioned study on individuals with substance use disorders (Flatscher-Bader et al., 2008). Overall, the present results partly support the hypothesis that glutamatergic signatures might emerge already in the initial stage of alcohol, but not nicotine, moderate intake. In regards to nicotine, the findings might suggest that, either the increased *VGLUT2* expression in human tobacco smokers was present prior to use, or that more extended life-long use is needed to increase *VGLUT2* expression.

Individual *Vglut2* subpopulations within the VTA have been suggested to play a unique role in drug addiction (Bimpisidis and Wallen-Mackenzie, 2019, Eskenazi et al., 2021; Morales & Root, 2014), with *Vglut2*-only neurons being implicated in both rewarding (Wang et al., 2015) and aversive (Root et al., 2018) behavior. On the other hand, *Vglut2^pos^/Th^pos^* neurons, comprising about 10-30% of VTA neurons, project selectively to the medial shell of the Acb, where they primarily target medium spiny neurons, mediating reward (Kawano et al., 2006; Mingote et al., 2019). Overexpression of VGLUT2 in dopaminergic neurons is associated with increased glutamate and dopamine signaling by co-release as well as an enhanced packaging and release of dopamine through *Vglut2* (El Mestikawy et al., 2011), and might be one of the underlying mechanisms leading to reinforcing effects. The present study, however, found some evidence supporting the involvement of the lateral aVTA *Vglut2-*only or *Vglut2^pos^/Th^pos^* in adolescent alcohol-, but not nicotine-related, aversive effects (Qi et al., 2016, Root et al., 2014). This is in line with the observed higher *Vglut2-*only and lower *Vglut2^pos^/Th^pos^* number of neurons respectively, in the aVTA of alcohol-only treated rats.

Importantly, the present findings confirm the heterogeneous composition of the VTA in terms of glutamatergic and dopaminergic neurons (Yamaguchi et al., 2007; Yamaguchi et al., 2011) and reinforce the suggestion of specific individual functions of the VTA sub-nuclei (Morales & Margolis, 2017). For example, in the aVTA, although no effect of the drugs was seen in the sum (%) of all neuronal subpopulations, *Vglut2^neg^/Th^pos^* neurons were less in the alcohol-only compared to the control group, a finding that indicates a potential aversive alcohol-related effect mediated by the aVTA. On the other hand, in the pVTA, the sum (%) of all cells was affected by nicotine administration, which was driven by the *Th*-only population. Indeed, *Vglut2^neg^/Th^pos^* neurons in the pVTA were more abundant in the group exposed to nicotine-only (corroborating evidence is presented as supplement). This finding supports the reinforcing effect of addictive drugs, especially nicotine, in the pVTA, being likely mediated by an increase in dopaminergic-only neurons (Romoli et al., 2019). Nonetheless, upon combination of both drugs, no effect on the *Vglut2^neg^/Th^pos^* neurons was observed in the total pVTA. However, in the lateral pVTA (i.e. PN), a lower number of *Vglut2^neg^/Th^pos^* neurons was seen in the combined-drugs compared to nicotine-only group. This may be due to interactive effects between the two drugs leading to distinct neuroadaptations upon co-use (Waeiss et al., 2019), possibly related to the drugs’ cross-tolerance (Funk et al., 2006). Moreover, dopaminergic neurons project from the lateral VTA to the lateral Acb shell and core, where dopamine release is not that robustly associated with reward, contrarily to the medial Acb shell (Ikemoto, 2007, Pontieri et al., 1995).

Methodological specifics should be considered when interpreting the present results. Outbred Wistar rats were used to mirror the heterogeneous genetic background among humans. As only male rats were included in the study, it remains to be investigated whether the present findings apply to females. *Truitt et al.* had previously shown altered *Vglut1-3* gene expression in the Acb of female alcohol-preferring rats upon acute administration of both alcohol and nicotine in the pVTA, while male rats had not been assessed (Truitt et al., 2015). Sex differences in both alcohol and nicotine reward-related behaviors exist, likely interacting with gonadal hormone fluctuations, and modulating both the glutamate and the dopamine systems (Becker & Koob, 2016). During adolescence, sex-specific effects of nicotine exposure have been consistently shown, whereas for alcohol, sex differences are still conflicting and could be species- or administration route-specific (Thorpe et al., 2020), calling for further research on both sexes. Subcutaneous nicotine injections and alcohol administration via gavage cannot be translated into a human way of smoking or drinking, respectively, but they minimize interindividual variations in drug intake; nonetheless, forced administration can represent a stressor. Moreover, the weekend-like drug administration regimen during PNW4 – 9 was modelled to mimic episodic drinking patterns seen in adolescents and young adults, albeit adding a component of potential withdrawal/negative affect during the drug-free days (Koob, 2021). Important to note is that the rats in the current study were exposed to pure nicotine, whereas cigarette smokers in the study of *Flatscher-Bader et. al* were exposed to a host of tobacco ingredients. Various additives in cigarettes e.g. menthol, vanillin, pyrazines and acetaldehyde, can affect the addictiveness of cigarettes by either direct interaction with nicotine, enhancing nicotine delivery, or by creating additional conditioned stimuli by chemosensory effects (Ahijevych & Ford, 2010; Alpert et al., 2016; Rabinoff et al., 2007). Furthermore, the molecular underpinnings leading to an addictive phenotype are the effect of a long process, whereas the present experimental model did not include animals showing signs of addiction, which makes very distinctive alterations, as the ones observed in post-mortem human brains (Flatscher-Bader et al., 2008), unlikely. It is possible that higher and more frequent doses for prolonged time would lead to different results. Last but not least, the animals were sacrificed two hours after the drug administration, thus the possible confounding effect of acute intoxication cannot be excluded. Nonetheless, it was within the interest of the present study to examine the acute nicotine - and/or alcohol-induced effects in a brain that has been protractedly exposed during adolescence, focusing on the differences between the two substances alone and in combination compared to the controls.

A strength of using dFISH and CISH/FISH targeting *Th* on the same slide, is the precise localization, rendering comparison between the groups within the VTA subregions highly accurate. Based on the functional antero-posterior heterogeneity of the VTA (Morales & Margolis, 2017; Sanchez-Catalan et al., 2014), the difference between the number of cells in each treated group vs. the control was assessed separately in each sub-region of the aVTA and the pVTA. Nonetheless, the present findings only apply to the selected bregma coordinates [i.e., -4.92mm (aVTA) and -5.3/-5.4mm (pVTA)], as the *Th* expression differed distinctly along the rostro-caudal axis, especially in the aVTA. More posterior parts of the VTA were not targeted as *Vglut2* expression is very low caudally to -5.5mm (Yamaguchi et al., 2011). Moreover, the standardized pipeline provided unbiased, automated image analysis of dFISH images (Carpenter et al., 2006), controlling for potential misidentification of cells. In the pVTA, manual counting was applied due to technical issues (i.e., very low staining quality), as well as two different imaging channels (i.e. brightfield and fluorescent). The counting was performed thrice, in a blind manner, following the same criteria of signal identification as in the automated analyses (i.e., staining was defined only if observed against a DAPI-stained nucleus). Lastly, though the *Th* marker is expressed only in neurons (Pickel et al., 1975) and not in astrocytes, our study cannot discard the possibility that astrocytic *Vglut2* expression contributes to the number of *Vglut2^pos^/Th^neg^* cells seen herein. Nonetheless, astrocytic *Vglut2* expression in the VTA is dubious (Cahoy et al., 2008; Li et al., 2013) and thus its contribution, if any, to the current findings should be negligible. Finally, nominally significant results were reported as only the control group was tested repeatedly.

## 5 Conclusions

The present study provides preliminary evidence on the effect of nicotine and alcohol during the crucial phase of adolescence on the ratio of glutamatergic and dopaminergic neurons in the VTA of male rats. The relative number of *Vglut2*-only or *Vglut2^pos^/Th^pos^-*expressing neurons was affected only in the aVTA upon repeated alcohol, but not nicotine, intake during adolescence. On the other hand, the relative number of *Th*-only positive neurons was lower in the aVTA of the alcohol-only group, but higher in the pVTA of the nicotine-only groups, suggesting a potential reinforcing effect of nicotine-only mediated by dopaminergic neurons in the posterior part of the VTA. The present findings support the hypothesis that VTA glutamate and dopamine-related signatures behind prolonged episodic adolescent exposure to the two most highly consumed substances, already emerge in the initial stage of use. Future studies should include more quantitative approaches on the cellular level to validate these findings and disentangle the heterogeneous functions of VTA *Vglut2* and *Th* neuronal populations that might contribute to the distinct effects found herein, in the anterior and the posterior VTA.

## Authors contributions

IN, EC, KWN, ÅWM and RB were responsible for the study concept and design. ALS performed the animal experiment. ALS and MV contributed to the acquisition of data. SD performed the FISH analysis. MV and ST performed the data analysis and interpretation of findings. MV, ST and EC drafted the manuscript. ÅWM and KWN provided critical revision of the manuscript for important intellectual content. All authors critically reviewed content and approved the final version for publication.

## Declaration of Competing Interest

The authors declare that they have no known competing financial interests or personal relationships that could have appeared to influence the work reported in this paper.

## Appendix Supplementary materials

Image, application 1
