# Supplementary material for: Vesicular glutamate transporter 2 expression in the ventral tegmental area of outbred male rats following exposure to nicotine and alcohol
Source: Drug Alcohol Depend Rep. 2023 Jul 13;8:100180. doi: 10.1016/j.dadr.2023.100180 (PMC10391930; doi:10.1016/j.dadr.2023.100180)
Supplement: Supplementary file 1 [file mmc1.docx]

***Vesicular Glutamate Transporter 2 expression in the ventral tegmental area of outbred male rats following exposure to nicotine and alcohol***

Maria Vrettou^a^, Stefan Thalhammer^a^, Anne-Lie Svensson^b^, Sylvie Dumas^c^, Kent W Nilsson^d^, Åsa Wallén-Mackenzie^e^, Robert Fredriksson^b^, Ingrid Nylander^b^, Erika Comasco^a^

1. Department of Women’s and Children’s Health, Science for Life Laboratory, Uppsala University, Uppsala, Sweden
2. Department of Pharmaceutical Biosciences, Uppsala University, Uppsala, Sweden
3. Oramacell, Paris, France
4. Centre for Clinical Research Västerås, Uppsala University, Västmanland County Hospital Västerås, Sweden
5. Department of Organismal Biology, Uppsala University, Uppsala, Sweden

**Supplementary material**

Effect of ethanol and/or nicotine exposure on *Vglut2-*expression and *Vglut2 /Th* neuronal subpopulations

In the pVTA, there was an interactive effect between nicotine and alcohol on the ratio (%) of *Vglut2neg/Thpos* neurons (F (1,21) = 6.118; *p* = 0.022). The ratio (%) of *Vglut2neg/Thpos* neurons was higher in the group exposed to nicotine-only (*p* = 0.019), tendentially higher in the group exposed to alcohol-only (*p* = 0.079), whereas no effect of combination of both drugs was observed.

The same effect was seen in the pPBP (F (1,21) = 6.924; *p* = 0.016), driven by the higher number (%) of *Th-*only positive neurons upon nicotine (*p* = 0.043) or tendentially higher *Th-*only neurons (%) upon alcohol exposure (*p* = 0.073) compared to controls, and a trend towards lower (%) *Th*-only positive neurons upon combination of drugs (*p* = 0.079) compared to nicotine-only group.


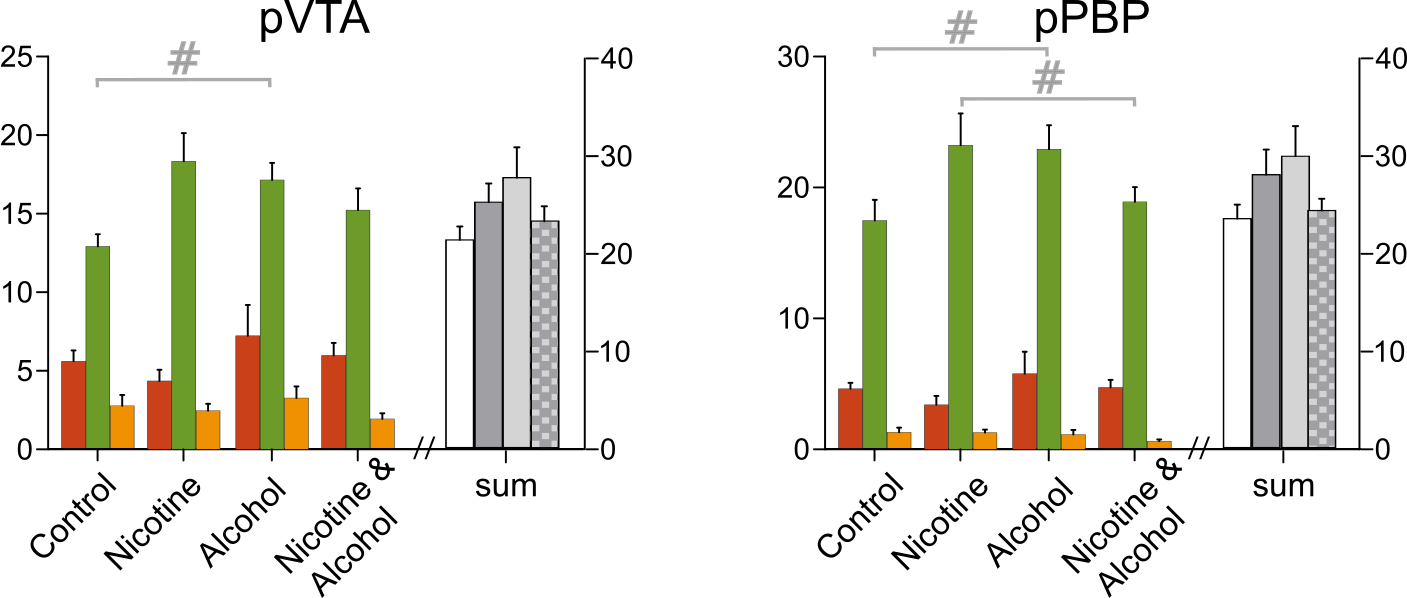


**Supplementary Figure 1.** Percentages of Vglut2pos/Thneg, Vglut2neg/Thpos, and Vglut2pos/Thpos neurons, and their sum, in the a) aVTA and PBpP among groups. Percentages of each neuronal population were estimated by using the count of neurons expressing Vglut2 and/or Th to the total number of the associated DAPI-stained nuclei within a region of interest

#: p ≤ 0.08; error bars = 1SE (standard error)

pVTA: posterior ventral tegmental area (VTA),; pPBP: posterior parabrachial pigmented nucleus

In the VTAR of the alcohol group there was a trend towards a lower percentage of *Th-*only neurons compared to the controls (*p =* 0.068). In the VTAR (*p* = 0.057) and aPBP (*p =* 0.072) of the alcohol group there was also a trend towards a lower percentage of *Vglut2pos/Thpos* neurons (VTAR: 1.4 ± 1.1; aPBP: 2.9 ± 1.3) compared to the control (VTAR: 3.3 ± 2.5; aPBP: 4.3 ± 2). A lower number of *Vglut2pos/Thpos* neurons was also seen in the aPBP of the combined group compared to nicotine-only group (See Figure below). On the other hand, a higher number of *Vglut2pos/Thpos* neurons has been found after adult nicotine exposure in mice that were neonatally exposed to nicotine (Romoli et al., 2019). Overexpression of VGLUT2 in dopaminergic neurons is associated with increased glutamate and dopamine signaling by co-release as well as an enhanced packaging and release of the dopamine through Vglut2 (El Mestikawy et al., 2011), and might be one of the underlying mechanisms leading to reinforcing effects. Herein, the tendentially lower percentage of *Vglut2pos/Thpos* neurons in the aPBP upon combination of the drugs could further support the aversion hypothesis (Qi et al., 2016; Root et al., 2014) mediated by the lateral aVTA in our model. Dopaminergic neurons from the lateral VTA project to the lateral Acb shell and core, where dopamine release is not that robustly associated with reward, contrarily to the medial Acb shell (Pontieri et al., 1995). Furthermore, most of the VTA neurons transiently express *Vglut2* during development, (aka before adulthood) (Dumas & Wallén-Mackenzie, 2019; Steinkellner et al., 2018). Thus, the effects seen herein could be a compensatory mechanism to the drug administration during the sensitive adolescent period.


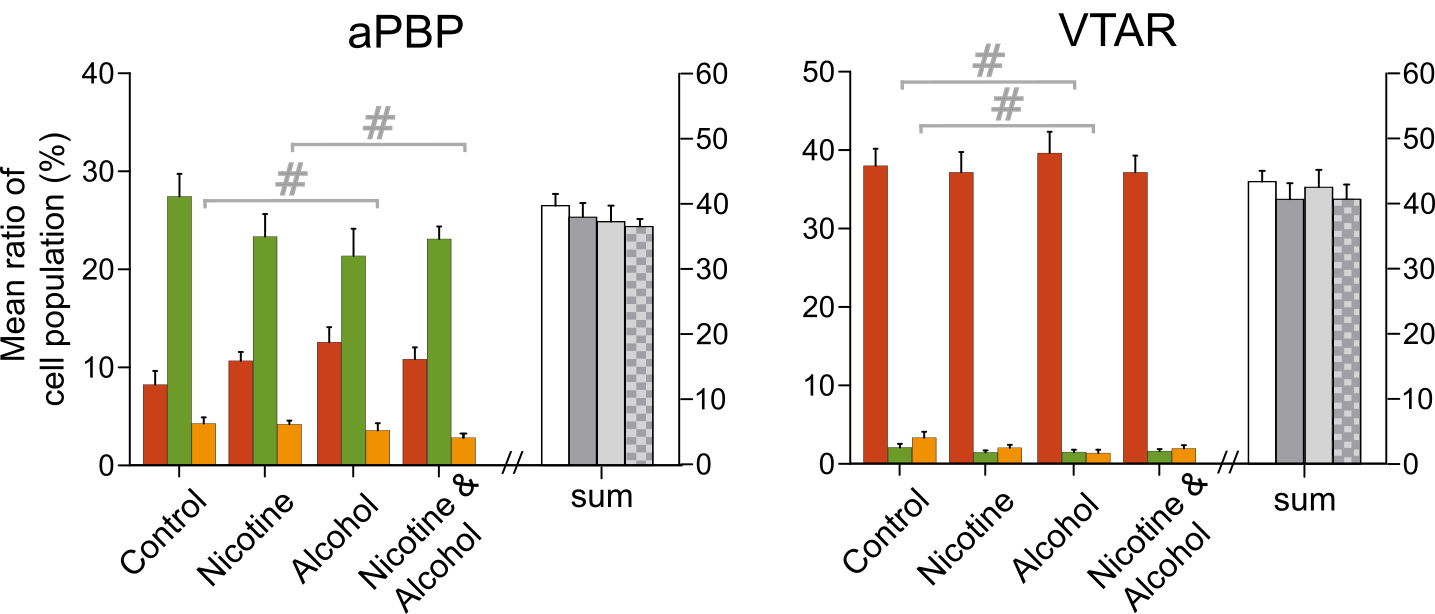


**Supplementary Figure 2.** Percentages of Vglut2pos/Thneg, Vglut2neg/Thpos, and Vglut2pos/Thpos neurons, and their sum, in the a) aPBP and VTAR among groups. Percentages of each neuronal population were estimated by using the count of neurons expressing Vglut2 and/or Th to the total number of the associated DAPI-stained nuclei within a region of interest

#: p ≤ 0.08; error bars = 1SE (standard error)

aPBP: anterior parabrachial pigmented nucleus; VTAR: VTA rostral nucleus

Interactive effect between region and group

GLM multivariate interaction model assessed whether there is an interactive effect between region (applicable only for the whole aVTA and pVTA) and group (control vs drug-treated groups) on the three different neuronal subpopulations. We found evidence for interaction between the group and the region on the Vglut2neg/Thpos population (F_(3, 51)_ = 4.266; p =0.009; partial eta square = 0.201; adj R square = 0.481). In the aVTA the drug-treated groups had lower number (%) of Vglut2neg/Thpos compared to the controls. The opposite pattern was observed in the pVTA, where the drug-treated groups had higher number (%) of Vglut2neg/Thpos compared to the controls. We didn’t find any significant interaction between group and region on the other two cellular subpopulations (Vglut2pos/Thneg, Vglut2pos/Thpos). These results (also graphically shown below) suggest region specific effects of ethanol and/or nicotine restricted to the Vglut2neg/Thpos population, complementing the findings of the analyses already presented in the manuscript.


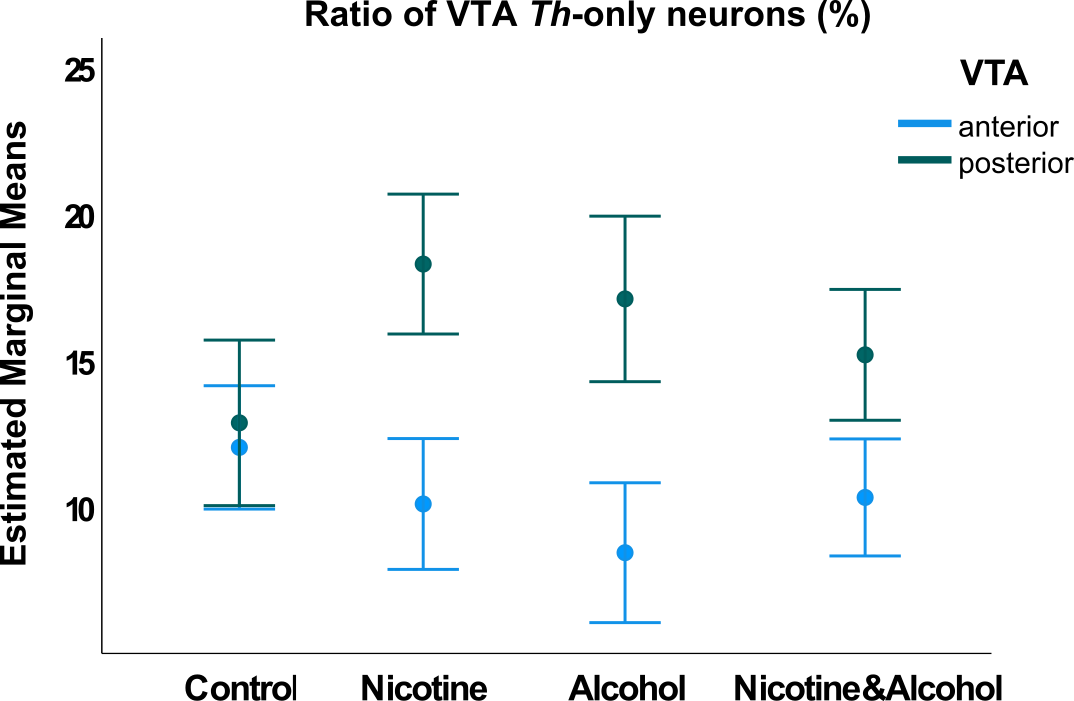


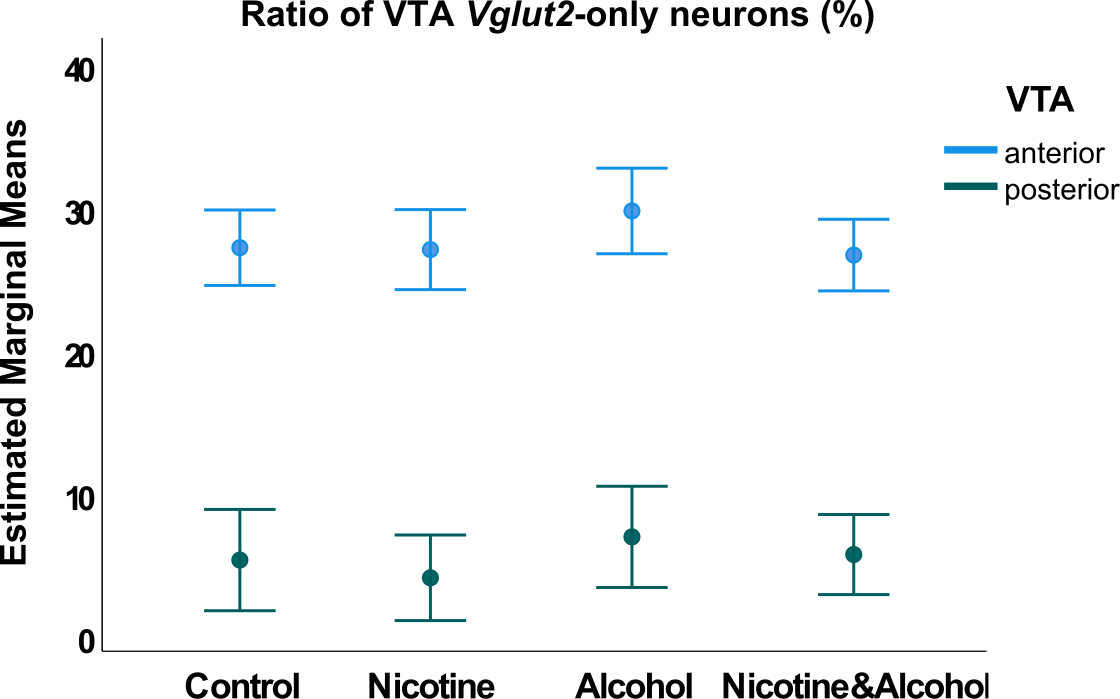


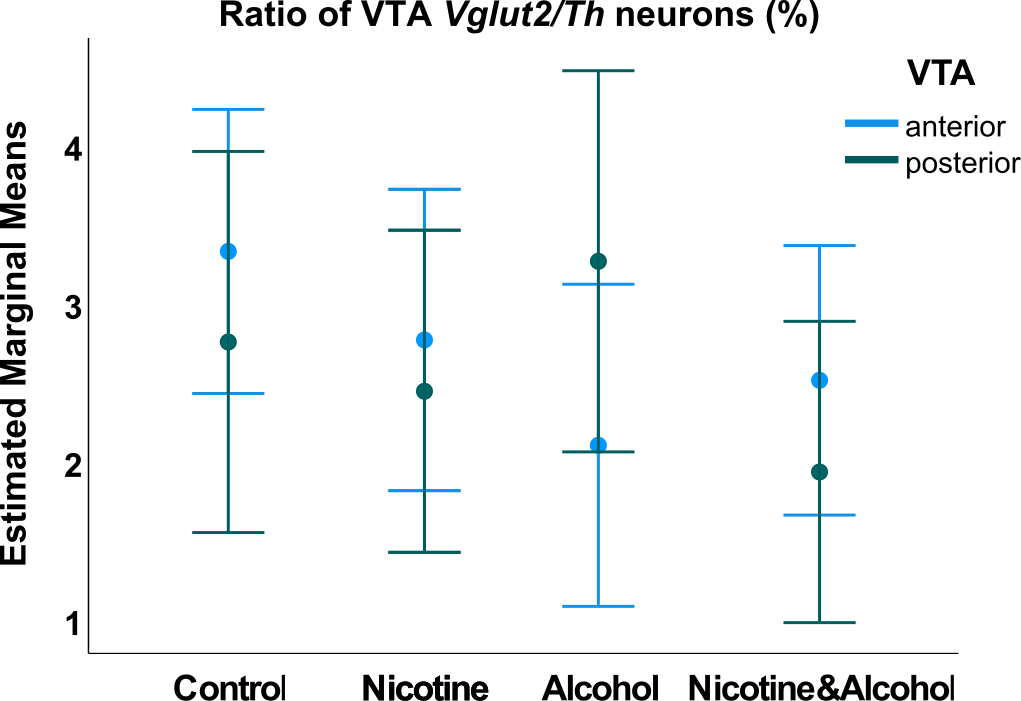


**Supplementary Figure 3.** Depiction of the interaction between region; i.e. the anterior (blue) and the posterior (green) VTA and group on the percentage of a) Vglut2neg/Thpos neurons, b) Vglut2pos/Thneg and c) Vglut2pos/Thpos. Error bars represent +/-1SE (standard error)
